# Kyphectomy with anterior column reconstruction using titanium mesh cage in meningomyelocele patients

**DOI:** 10.1051/sicotj/2022006

**Published:** 2022-03-07

**Authors:** Mohammed Ali Hussien, Ahmed Elbadrawi, Mohammed Zayan

**Affiliations:** Department of Orthopedic Surgery, Faculty of Medicine, Ain Shams University 11591 Cairo Egypt

**Keywords:** Myelomeningocele, Kyphosis, Kyphectomy, Titanium mesh cage

## Abstract

*Study design*: Prospective case series. *Purpose*: To describe a new technique for anterior column reconstruction after kyphectomy in myelomeningocele patients using titanium mesh cage and to evaluate outcomes and complications. *Methods*: Sixteen patients with severe dorsolumbar kyphosis 2^ry^ to myelomeningocele were enrolled with a mean age of 10.1 years. Kyphectomy procedure and long spinopelvic fixation were done, titanium mesh cage was used to reconstruct the anterior column. Operative time and intraoperative blood loss were calculated. Using the Cobb method, pre and postoperative measurements of local/regional kyphosis were done. Degree and mean percentage of correction were calculated. Anterior intervertebral height of the kyphotic area was also measured. The mean follow-up period was 27 months. *Results*: Operative time was 271.3 min ± 25, and estimated intraoperative blood loss was 781.3 mL ± 92.3. On average, 2.5 vertebrae were resected. All 16 patients were able to lie supine immediately postoperatively. The mean preoperative local/regional kyphosis was 107.5°, and 106.9° respectively, corrected to 22.5° and 28.8° postoperatively, with a mean degree of correction of 85° and 78.1° respectively. Mean preoperative anterior intervertebral height was 3.54 cm, improved to 4.64 cm postoperatively. Only 2 cases had a superficial wound infection managed conservatively. At the latest follow-up, no loss of correction pseudoarthrosis occurred, and all patients showed solid fusion. *Conclusion*: Titanium mesh cage is an efficient, easy method for anterior reconstruction following kyphectomy in myelomeningocele patients, to maintain postoperative correction.

**Level of evidence:** Therapeutic studies, Level IV study

## Introduction

Severe rigid progressive kyphosis is a challenging problem in myelomeningocele patients. Typically, lumbar or dorsolumbar junction is affected and usually associated with postural problems. A kyphotic deformed spine is present in 8–20% of these patients [1, 2]. This causes severe postural problems, inability to lie supine, pressure sores over the apex increasing the infection risk [1], and progressive loss of the ability to sit unassisted, which limits bimanual functions being used for truncal upright support [1]. Most curves are rigid, often exceed 80° at birth, and yearly progress by 6° to 12° to reach severe degrees (140° or more) [3]. Having unique features and comorbidities, the goals of and indications for surgery are different from other deformity patients where the primary surgical goal is the restoration of the child’s sitting balance [3, 4]. The secondary goals of surgical correction are to stop deformity progression, increase stability and halt cardiopulmonary deterioration. Surgical correction is therefore inevitable in established progressive kyphosis and is now well documented since the introduction of the kyphectomy technique by Sharrard [5]. Garg et al. [3] and Sponseller et al. [4] have shown functional benefits for the patients and improvements in sitting position, quality of life, and pulmonary function. Different techniques have been described for correction and long spinal stabilization; Traditionally, autografts from resected vertebrae have been used for anterior reconstruction after kyphectomy. However, this autograft alone without anterior strut is associated with high rates of pseudoarthrosis and correction loss, still posing a major unresolved problem [6]. The purpose of this study is to develop a reliable and safe method for anterior reconstruction in these patients using the titanium mesh cage (TMC), which is an attractive alternative, being versatile in the shape, diameter and length, also being hollow to accommodate morselized bone graft [7].

To the best of the author’s knowledge, at the time of writing, there is no published report in the English literature about using Titanium mesh cage as a post kyphectomy anterior column reconstruction in myelomeningocele patients.

## Materials and methods

Sixteen myelomeningocele patients with severe rigid kyphosis were enrolled in this prospective study, patients mean age was 10.1 years ± 1.7. Ten females (62.5%), and six males (37.5%). The follow-up period ranged from 25 to 28 months (mean 27 months) (Table 1).

**Table 1:** Demographic data, resected vertebra, follow-up period, operative time, and blood loss.

Patient	Sex	Age	Resected vertebra	Follow-up (month)	Operative time (min)	Blood loss (mL)
1	Male	9	L1, L2	26	240	750
2	Female	10	D12, L1, L2	28	300	800
3	Male	12	D11, D12	19	270	850
4	Female	12	D11, D12, L1	27	300	750
5	Male	8	D12, L1, L2	20	280	900
6	Female	8	D12, L1, L2	28	290	850
7	Female	12	D12, L1	18	250	750
8	Female	10	D11, D12	26	240	600
9	Female	10	D12, L1, L2	28	300	800
10	Male	9	L1, L2	26	240	750
11	Female	12	D11, D12, L1	27	300	750
12	Male	12	D11, D12	19	270	850
13	Female	8	D12, L1, L2	28	290	850
14	Male	8	D12, L1, L2	20	280	900
15	Female	10	D11, D12	26	240	600
16	Female	12	D12, L1	18	250	750

Inclusion criteria included wheelchair-bound myelomeningocele patients, progressive kyphosis, inability to lay down in the supine position, difficult balanced sitting, and recurrent skin problems over the gibbus.

Exclusion criteria included deformities other than kyphosis, previous failed kyphectomy attempts, active skin infection over the gibbus, and significant comorbid medical conditions.

Preoperative assessment for each patient in this study was carefully done clinically via a detailed history thorough general and local examination. The age, sex, chief complaint, skin condition, and the curve pattern of the patients were recorded. All patients were wheelchair-bound, with no neurologic function distal to their gibbus and a thoracic sensory level. Significant focus was placed on an assessment by anesthesiologists to identify fitness and prerequisites for surgery.

**Figure 1:**
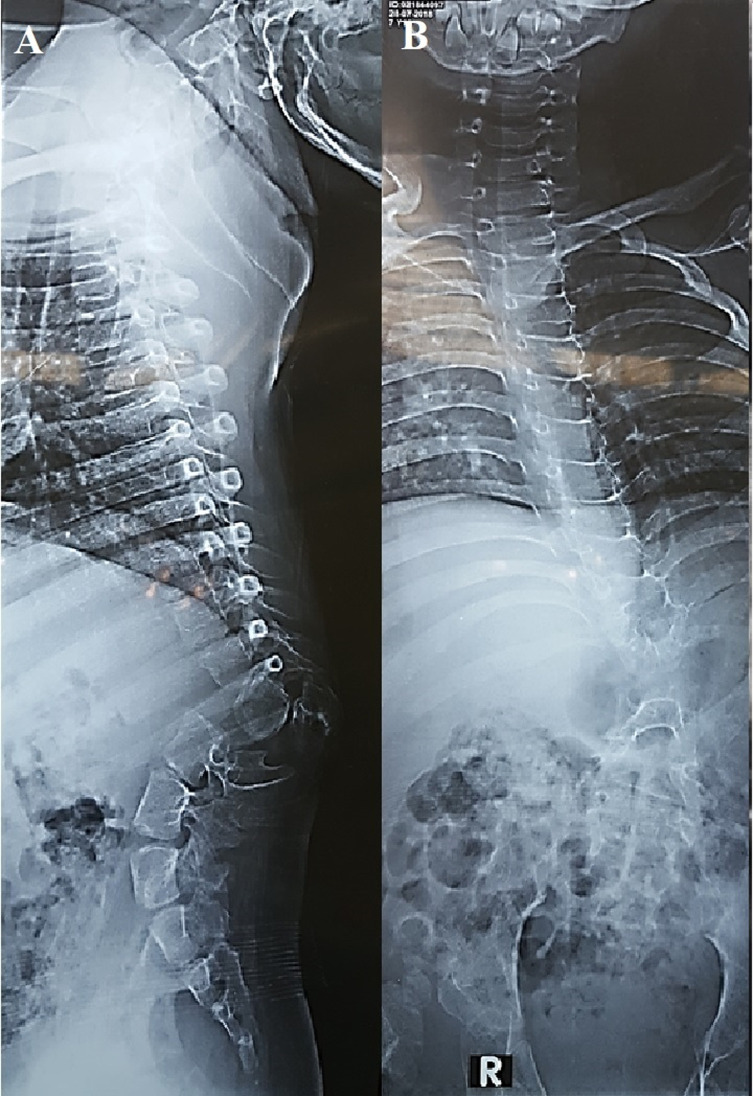
Preoperative. (A) Lateral plain X-ray showing acute kyphosis. (B) Anteroposterior plain X-ray showing coronal deformity.

Radiographic evaluation was performed using sitting whole spine anteroposterior and lateral plain radiographs (Figure 1), kyphosis was classified according to its region, and curve apex was noted. Preoperatively and immediately postoperatively, local kyphosis was measured using Cobb’s method, and regional sagittal alignment (RA) for the dorsolumbar area was also assessed. The anterior intervertebral height of the kyphotic area was calculated as the vertical height between anterior endpoints of the superior endplate of the vertebra proximal and distal to the kyphectomy site. Preoperative CT scans with 3D reconstruction images were obtained for better preoperative planning. MRI was performed to search for additional neural axis anomalies. Operative data included mean operative time, estimated blood loss, number and location of resected segments, and intraoperative complications.

Postoperatively, no thoraco-lumbo-sacral orthoses were used. All patients we able to lay supine in bed immediately postoperatively, and resumption of daily activities was allowed as tolerated.

### Follow-up protocol

**Figure 2:**
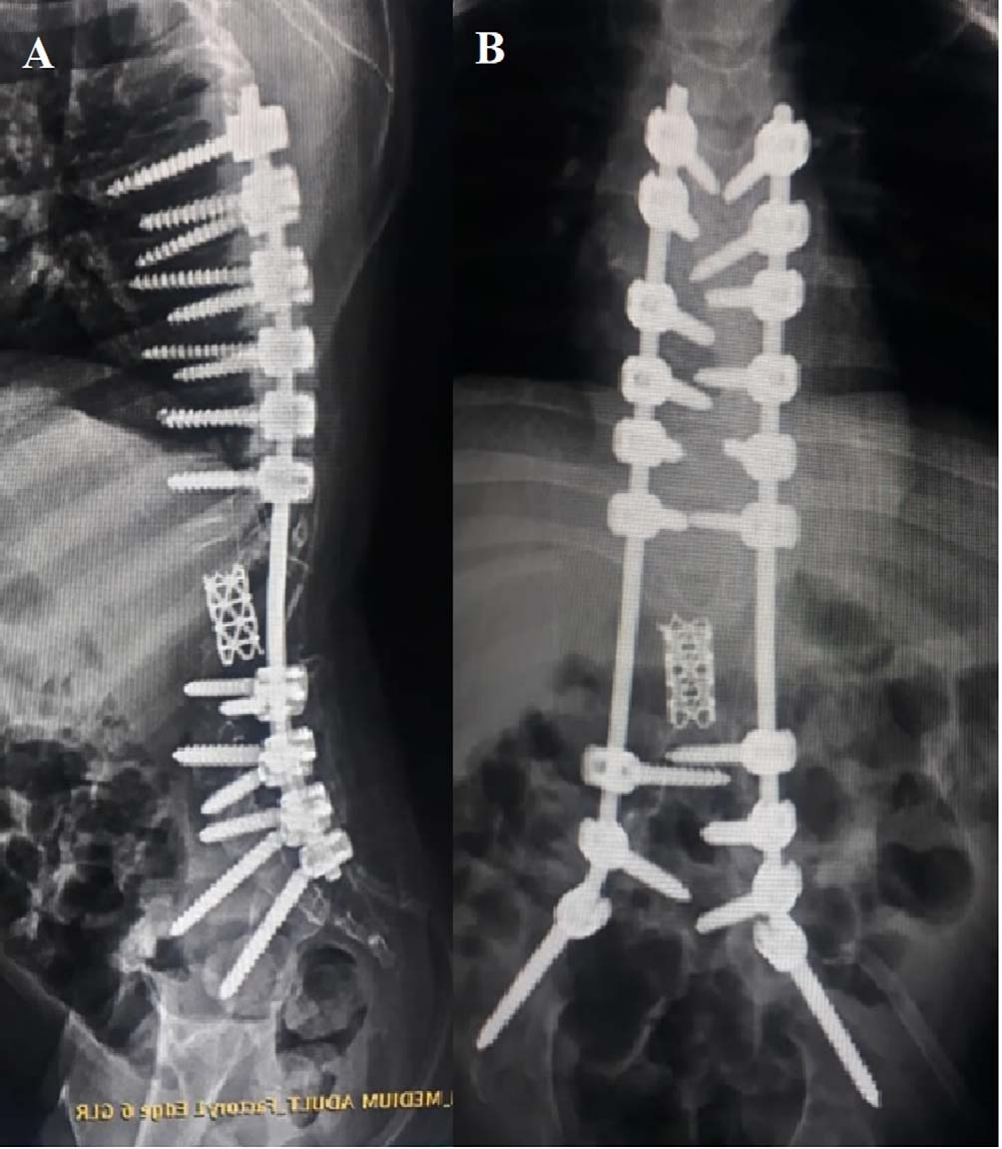
Postoperative. (A) Lateral plain X-ray showing kyphectomy/titanium mesh cage inserted. (B) Anteroposterior plain X-ray showing S2 alar iliac screws/titanium cage.

**Figure 3:**
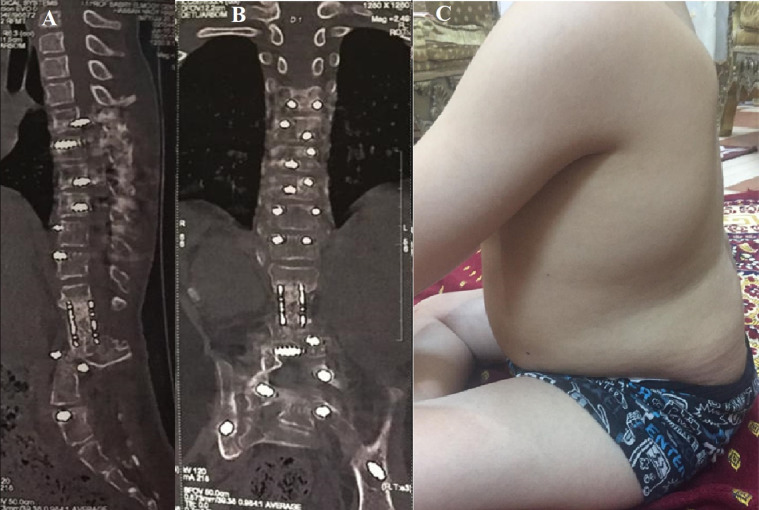
Last follow-up showing continuous bony bar inside cage. (A) Sagittal CT scan. (B) Coronal CT scan. (C) Clinical lateral photo showing sagittal profile.

After their discharge, patients were subsequently seen for follow-up in the outpatient clinic at 2 weeks to monitor incision healing, then at 1, 3, 6, 12, and 24 months with whole spine radiographs (Figure 2). Assessment of the construct position and checking for fusion mass, implant failure, mesh cage subsidence, and loss of correction on plain radiographs were made at each visit, and complications were recorded. Fusion was regarded to be attained once a solid sheet of freshly shaped bone was clear along the rods or when a distinct bony bridge through the mesh cage was observed on a plain radiograph or CT scan (Figure 3). CT scan with 3D reconstruction images was used only if solid fusion was suspected but inconclusive.

### Surgical technique

All patients were surgically treated via kyphectomy and long spinopelvic fixation using only pedicle screws and S2 Alar iliac (S2AI) screws combined with the proposed technique of titanium mesh cage reconstruction of the defect between January 2017 and January 2019.

Following prone positioning and prophylactic antibiotic administration, the skin incision is fashioned according to the patient’s skin condition, mostly a longitudinal incision at the area of normal spinal and careful development through the site of previous sac closure. Skin adherences were dissected meticulously to preserve the skin vitality, skin flaps were developed as thick as possible. Subperiosteal elevation of paraspinous muscles using electrocautery exposing the transverse processes was done starting proximally from the normal level. Fluoroscopically assisted, Pedicle screws were inserted using the standard anatomical landmarks in the proximal segments with at least six secure fixation points. As for the distal segments, residual vertebrae remaining distal to the planned resection determined the number of fixation points, mostly, in addition to the S2AI screws, at least 2 vertebrae were instrumented from L4 to S1. Thus, assuring at least six rigid fixation points distally. The midpoint between the S1 and S2 foramina was used as an entry point for S2AI screws and the trajectory was angled 40° laterally and 40° caudally aiming towards the AIIS.

At the planned kyphectomy segment, careful dissection ligation of the segmental vessels and nerves was done. Subperiosteal blunt dissection using surgical sponges aided by bipolar electrocautery is continued laterally and anteriorly from the transverse processes, circling around the vertebral bodies till the midline. At this point, several fingers or a blunt dissector can be passed freely anterior to the kyphotic bodies. The kyphotic deformity can now be clearly viewed.

Kyphectomy started by removing the transverse processes and abnormal pedicles, followed by the lateral aspects of the vertebral bodies, lastly, the apex is removed using curved curettes and rongeurs through the disc spaces or the bodies in a piecemeal fashion. Disc levels are carefully identified to allow endplate preparation. Rods are contoured and secured to distal anchorage points, then corrected by cantilevering the rods onto the proximal anchorage screws, rotating the pelvis, and restoring sagittal alignment.

**Figure 4:**
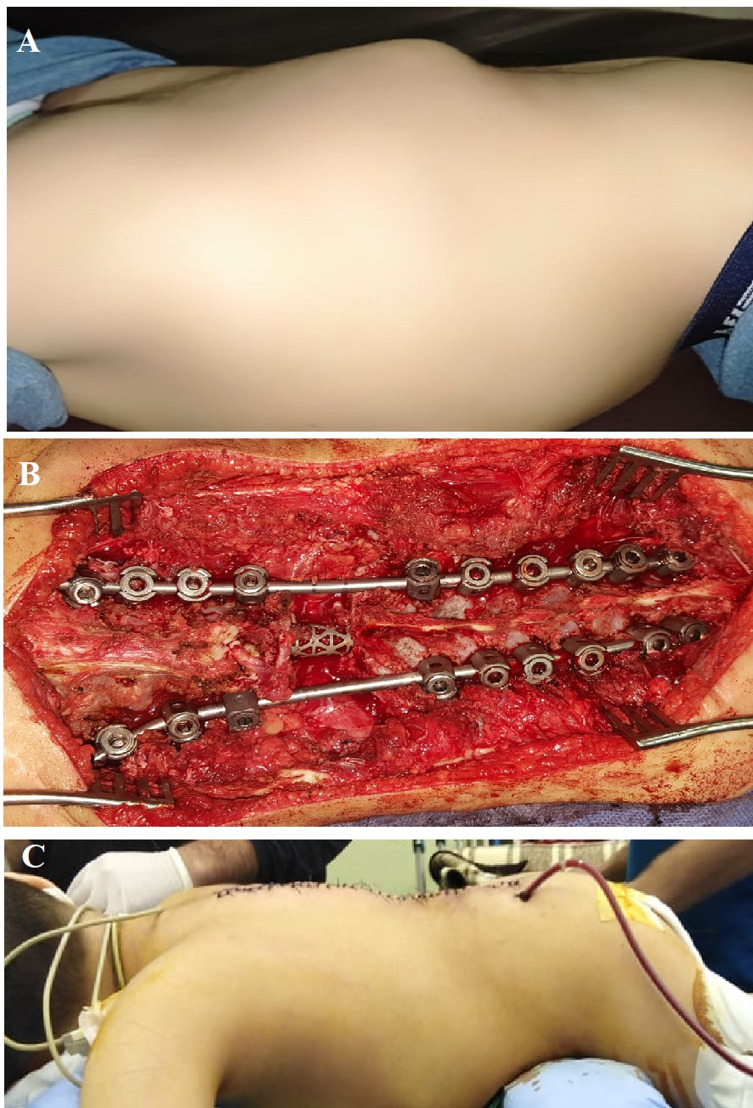
Clinical photos. (A) Preoperative kyphus. (B) Intraoperative correction (screws + titanium cage). (C) Postoperative correction.

The anterior column gap produced by resection was reconstructed using a non-expandable titanium mesh cage. After measuring the needed size and length, an appropriate cage “filled with morselized bone from the resected body” is impacted guided by fluoroscopy, and subsequent compression is done to avoid dislodgment (Figure 4). Posterior decortication and morselized bone graft are inserted along with the whole instrumentation levels.

Soft tissue closure was performed in layers. Skin and subcutaneous tissue were abundant after flattening of gibbus, spine realignment, and relative shorting.

## Results

The mean operative time in this study was 271.3 min ± 25.3. The mean operative blood loss was 781.3 mL (range 600–900 mL) (Table 1).

A number of resected levels were two-level resections in 50%, and three-level resections in 50%.

On average, 2.5 vertebrae were resected. Reconstruction was done by titanium mesh cage in all patients.

The preoperative local kyphosis (Cobb’s angle) ranged from 70° to 125° (mean 107.5°) that improved from 10° to 35° (mean 22.5°) postoperatively, with a mean correction of 85° (Table 2).

**Table 2:** Preoperative and postoperative kyphosis, percentage of correction, preoperative, postoperative, and last follow-up anterior vertebral heights.

Patient	Pre local kyphosis	Post local kyphosis	% local correction	Pre regional kyphosis	Post regional kyphosis	% regional correction	Pre anterior height (cm)	Post anterior height (cm)	Last follow up anterior height (cm)
1	70	10	85%	80	25	68%	3.2	4.2	4.2
2	125	30	76%	135	45	66%	3.9	5.1	5.1
3	125	35	72%	130	50	62%	4	5.2	5.2
4	110	20	82%	95	15	84%	3.3	4.4	4.4
5	115	20	83%	115	20	83%	3.5	4.5	4.5
6	120	25	79%	125	40	68%	3.4	4.6	4.6
7	100	25	75%	95	15	84%	3.7	4.9	4.9
8	95	15	84%	80	20	75%	3.3	4.2	4.2
9	125	30	76%	135	45	66%	3.9	5.1	5.1
10	70	10	85%	80	25	68%	3.2	4.2	4.2
11	110	20	82%	95	15	84%	3.3	4.4	4.4
12	125	35	72%	130	50	62%	4	5.2	5.2
13	120	25	79%	125	40	68%	3.4	4.6	4.6
14	115	20	83%	115	20	83%	3.5	4.5	4.5
15	95	15	84%	80	20	75%	3.3	4.2	4.2
16	100	25	75%	95	15	84%	3.7	4.9	4.9

The preoperative RA ranged from 80° to 135° (mean of 106.9°) and improved from 15° to 50° postoperatively (mean 28.8°) (Table 2).

**Figure 5:**
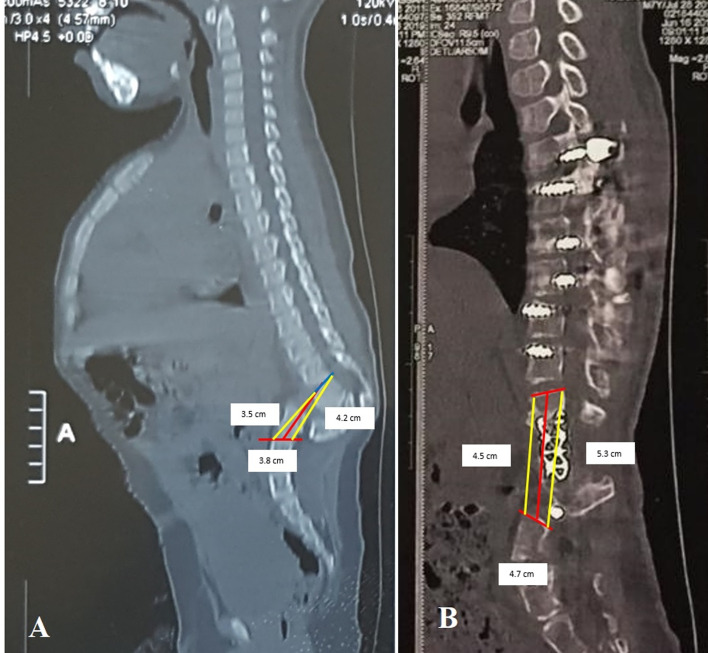
Anterior intervertebral height of kyphotic area. (A) Preoperative. (B) Postoperative.

The preoperative anterior intervertebral height of the kyphotic area ranged from 3.2 to 4 cm (mean 3.54 cm) and improved from 4.2 to 5.2 cm (mean 4.64 cm) postoperatively (Figure 5) (Table 2).

The improvements were all highly statistically significant (*P* < 0.001).

### Complications

No intraoperative complications occurred, postoperatively only two patients suffered from superficial wound infection and were managed conservatively.

At final follow-up, there was no implant failure, no pseudoarthrosis or loss of correction in the sagittal plane, and no cage subsidence.

## Discussion

Achieving a stable correction and attaining a solid fusion after kyphectomy is far more important than the degree of correction [8]. Not having anterior structural support with Posterior fusion puts fused segments under tension due to long bending moment, ultimately leading to implant failure [9]. Therefore, developing a reliable method for anterior column reconstruction is highly important.

Our results are highly comparable to previously published studies in the literature (Table 3) as regards overall correction, operative time, and blood loss, with better results regarding no loss of correction, pseudoarthrosis, or implant failure at the end of follow-up period. Our study is superior to previous studies done on kyphectomy technique, measuring the improvement in Anterior intervertebral height of the kyphotic area from a mean of 3.5 cm to a mean of 4.6 cm which was highly statistically significant (*P* < 0.001) and remained the same at the end of follow-up period denoting no loss of correction.

**Table 3:** Summary of previous studies compared to present study.

Study	Number of patients	Anterior reconstruction	Operative time (minutes)	Loss of correction	Complications
Brown et al. [10]	–	Fibula	–	–	–
Lindseth and Stelzer [27]	23	–	–	13	13/23
Lintner and Lindseth [28]	39	–	–	9	9/39
Fürderer et al. [29]	14	–	216	–	7/14
Odent et al. [11]	9	Tiba	–	None	3/9
Niall et al. [2]	24	–	285	8	20/24
Akbar et al. [1]	24	–	282	7	12/24
Garg et al. [3]	18	–	342	–	7/18
Comstock et al. [30]	22	–	248	9	10/22
Hussien et al. (present study)	16	Titanium mesh cage	271	None	2/16

In most published literature discussing kyphectomy in myelomeningocele, morselized graft alone from resected vertebrae have been used to reconstruct the anterior column [1, 2, 6]. Only two publications reviewed anterior reconstruction by struts. Brown [10] introduced a two-stage procedure during a symposium, including posterior kyphectomy with Dwyer internal fixation than a second stage anterior strut grafting by a fibula. Odent et al. [11] also reviewed a two-stage surgical procedure with first stage posterior kyphectomy using a modified Dunn–McCarthy fixation than a second anterior stage using the thoraco-abdominal approach to insert an inlay strut graft from T10 to S1. The authors stated that posterolateral grafting is not enough following kyphectomy and lack of adequate anterior fusion explains deformity recurrence and failure [11]. To the best of the author’s knowledge, no other published articles discussed anterior strutting following kyphectomy in myelomeningocele patients.

Numerous techniques are used for anterior column reconstruction following vertebral resection [12]. Conventionally, a strut autograft is used, either a resected rib, tricortical iliac, fibular strut, or allograft [13, 14]. No adequate stability is provided when using ribs. Being fragile, with high plastic deformation and a small contact area [14]. As for tricortical iliac struts, fashioning the shape and size is difficult, having relatively small weight-bearing surfaces also increased donor site morbidity [15, 16]. A strut with proper length is a challenge as typically, more than a single body is resected. Immobilization is another major concern till autograft incorporation occurs.

The TMC is a versatile, attractive alternative allowing intraoperative flexibility, especially with these large defects, being available in multiple diameters, which allows maximal contact, adjusted lengths customized to the defect, also being hollow, thus accommodating morselized bone graft acts as a bony conduit [13], it immediately supports the anterior column, providing resistance to rotation, axial compression, lateral flexion, and toggling in both the sagittal and coronal planes [16, 17]. The significant surface area interface prevents it from extrusion or displacement [18]. In 1996, Lowery and Harms [19] confirmed TMC’s outstanding biomechanical characteristics using a static peak load test and a cyclic endurance test after more than a million cycles. In another study done by Hollowell et al. [20], under axial loading, the TMC had greater resistance to subsidence than other struts. Non-expandable titanium cages are classically used for anterior column reconstruction in trauma, infection, or tumors, with reported high rates of clinical outcomes with very low implant-related problems [21]. Reported radiographic and histopathologic fusion with autologous bone grafts inside titanium mesh cages ranged from 47 to 100% [7, 21, 22]. This figure was better than the results reported by Bridwell et al. [23] and Buttermann et al. [24] using structural allograft with a fusion rate of 92% and 91.4%, respectively. However, despite numerous publications using TMC in infection, trauma, and tumors, there is no data on their application post kyphectomy anterior reconstruction in Myelomeningocele patients.

Around 80% improvement of kyphosis angle was achieved in this study, which is comparable to other series in literature as Ko et al. [25], de Amoreira et al. [26]. Mean operative time was 4.5 h which was shorter compared to 5.7 h in Garg et al. [3] and 4.7 h in Akbar et al. [1] studies.

It is difficult to compare complication rates between kyphectomy series, loss of correction occurred in many studies, Lindseth et al. [27] had 13 out of 23 patients, Linter et al. [28] had 9 out of 39 patients, and Fürderer et al. [29] had 7 revisions out of 14 patients. At the end of the follow-up period of this study, no single case had a loss of postoperative correction. No revision surgeries were done in our series; on the contrary, Garg et al. [3] reported 7 revisions out of 18 cases, Comstock et al. [30] reported 8 implant removals in their study. Two of the largest series in literature each reporting 24 cases had a high rate of complications were Niall et al. [2] reported 20 complications and Akbar et al. [1] reported 12 complicated cases including a perioperative death. Our only reported complication was a superficial wound infection managed conservatively.

Limitations of this prospective study were small numbers of patients, lack of a comparative group, and being a mono-centric study, this requires a future multi-centric study with a larger number of patients and a comparative group.

## Conclusion

This study demonstrates that Titanium mesh was effective at maintaining post kyphectomy correction over a follow-up period of 27 months.

## Conflict of interest

The authors declare that they have no relevant financial or non-financial interests to report.

## Funding

This research did not receive any specific funding.

## Ethical approval

The study was conducted after approval of the ethics committee of our institute (IRB No. FMASU 1967/2014).

## Informed consent

This prospective case study was approved by the institutional review board of the faculty of medicine Ain Shams University in accordance with the ethical standards of the institution and national research committee and with the 1964 Helsinki declaration and its later recommendations or comparable ethical standards. Informed consent was obtained from all patients included in this study for participating and publishing of this study. All steps and details of the procedures were explained to the participants.

The participant has consented to submitting the case report to the journal.

The authors affirm that human research participants provided informed consent for the publication of the images.

Written informed consent was obtained from all patients and/or families.

## Author’s contributions

All authors contributed to the study’s conception and design. Material preparation, data collection, and analysis were performed by Mohammed Hussien and Ahmed Elbadrawi. The first draft of the manuscript was written by Mohammed Hussien, and all authors commented on previous versions of the manuscript. All authors read and approved the final manuscript.

All authors declare no significant competing financial, professional, or personal interests that might have influenced the performance or presentation of the work described in this manuscript.

## Data availability

The datasets generated during and/or analyzed during the current study are available from the corresponding author on reasonable request.
